# The mitochondrial transcriptome of the anglerfish *Lophius piscatorius*

**DOI:** 10.1186/s13104-019-4835-6

**Published:** 2019-12-10

**Authors:** Arseny Dubin, Tor Erik Jørgensen, Lars Martin Jakt, Steinar Daae Johansen

**Affiliations:** Genomics Group, Faculty of Biosciences and Aquaculture, Nord University, 8049 Bodø, Norway

**Keywords:** Anglerfish, Antisense RNA, Humanin, Mitogenome, Long noncoding RNA, lncCOI, mtDNA

## Abstract

**Objective:**

Analyze key features of the anglerfish *Lophius piscatorius* mitochondrial transcriptome based on high-throughput total RNA sequencing.

**Results:**

We determined the complete mitochondrial DNA and corresponding transcriptome sequences of *L. piscatorius*. Key features include highly abundant mitochondrial ribosomal RNAs (10–100 times that of mRNAs), and that cytochrome oxidase mRNAs appeared > 5 times more abundant than both NADH dehydrogenase and ATPase mRNAs. Unusual for a vertebrate mitochondrial mRNA, the polyadenylated COI mRNA was found to harbor a 75 nucleotide 3′ untranslated region. The mitochondrial genome expressed several non-canonical genes, including the long noncoding RNAs lncCR-H, lncCR-L and lncCOI. Whereas lncCR-H and lncCR-L mapped to opposite strands in a non-overlapping organization within the control region, lncCOI appeared novel among vertebrates. We found lncCOI to be a highly abundant mitochondrial RNA in antisense to the COI mRNA. Finally, we present the coding potential of a humanin-like peptide within the large subunit ribosomal RNA.

## Introduction

The mitochondrial genome (mtDNA) gene content and organization is highly conserved among vertebrates [1]. All species investigated to date encode the same 37 canonical gene products of 13 hydrophobic membrane proteins, 2 ribosomal RNAs (mt-rRNAs), and 22 transfer RNAs (tRNAs), as well as several non-canonical peptides and long noncoding RNAs (lncRNAs) [2]. The corresponding mitochondrial transcriptomes are less studied and have mainly been investigated in a small number of vertebrates including some mammalian cells and tissues [3, 4] and in gadiform fishes [5, 6]. Only minor differences were noted between the mammals and fish. In general, three polycistronic transcripts initiated from two H-strand promoters (HSP_1_ and HSP_2_) and one L-strand promoter (LSP) are involved in mitochondrial gene expression. Whereas the highly abundant HSP_1_ transcript mainly generates mt-rRNAs, the HSP_2_ transcript is responsible for most messenger RNAs (mRNAs) and tRNAs. The LSP transcript generates one mRNA and eight tRNAs.

Atlantic cod mt-rRNAs are oligo-adenylated [5], and fold into similar secondary structures as in other fish species [7, 8]. Interestingly, several mitochondrial-derived peptides (MDP) have been proposed to be encoded on both strands of the mt-rRNA gene locus [9], and two MDPs (MOTS-c and Humanin) have coding potential in Atlantic cod [2]. Mature tRNAs carry the non-template CCA at their 3′ ends and fold into the common tRNA patterns [7, 10]. Eleven mature mRNAs were found expressed in the Atlantic cod mitochondria, 10 from the HSP_2_ transcript and one from LSP, and two of the HSP_2_-specific mRNAs were bicistronic (ND4/4L and ATPase8/6) [6]. All mRNAs, except the LSP-specific ND6 mRNA, were found polyadenylated.

Mitochondrial lncRNAs have been identified and investigated in Atlantic cod [2]. Here, lncCR-H and lncCR-L correspond to different strands of the mitochondrial control region (CR). Both lncRNAs are clearly expressed and appear to generate small stable mitochondrial RNA (mitosRNA) [2, 6, 11, 12]. We recently reported low-level substitution heteroplasmy of the anglerfish *Lophius piscatorius* based on SOLiD deep sequencing [13]. As part of a study to generate a full reference genome and transcriptome for *L. piscatorius*, we here present the complete mitochondrial genome and key features of the corresponding mitochondrial transcriptome.

## Main text

### Methods

#### Nucleic acid extraction and high-throughput sequencing

*Lophius piscatorius* tissue samples were collected from two specimens obtained by commercial fishery off the coast of Nordland County, Northern Norway, in 2015 (BF1) and 2017 (BF2). Total DNA from BF1 was extracted from muscle tissue and sequenced by the SOLiD5500 and Ion PGM platforms as described previously [13]. Total DNA sequencing (head kidney) of BF2 using the Illumina HiSeqX platform was performed by Dovetail Genomics (Chicago, US) as a service [14]. Total RNA from heart muscle tissue of specimen BF2 was isolated using QIAzol Lysis Reagent (QIAGEN, Hilden—Germany) according to the manufacturers protocol. Cellular rRNA was depleted from 1 μg of total RNA using the RiboMinus™ Eukaryote System v2 (Thermo Fisher Scientific, Waltham, MA—USA), and whole transcriptome library was constructed using the Ion Total RNA-seq kit v2 (Thermo Fisher Scientific) according to the manufacturers protocols. Manual template preparation on an Ion OneTouch™ 2 System (Thermo Fisher Scientific) and sequencing of two Ion 540™ chips on the Ion GeneStudio™ S5 System (Thermo Fisher Scientific) were carried out at our Genomics Platform (Nord University) according to the manufacturers protocols. The sequencing resulted in a total of 154,741,088 reads with a mean read length of 169 nt, corresponding to 26 billion nt.

#### Data analysis

RNA reads were quality trimmed with Cutadapt [15] using q20 as a threshold. The minimum read length was set to 50 nt. Trimmed RNA reads were then mapped to the BF2 mitochondrial genome with CLC Genomics Workbench v12 (QIAGEN). The “Length fraction” parameter was set to 0.9 and “Similarity fraction” to 0.96, requiring at least 90% of the read length to map with 96% similarity. Other parameters were set to their defaults. The resulting BAM file was coordinate sorted with SAMtools [16] and then processed with BEDTools [17] (genomecov command) to obtain a base level coverage of the mitogenome. Mean coverage for each gene and non-coding region was calculated from bed file. Alignments were visually examined to identify non-coding RNAs and polyA tails.

### Results

#### *Canonical mitochondrial genes in L. piscatorius*

**Fig. 1:**
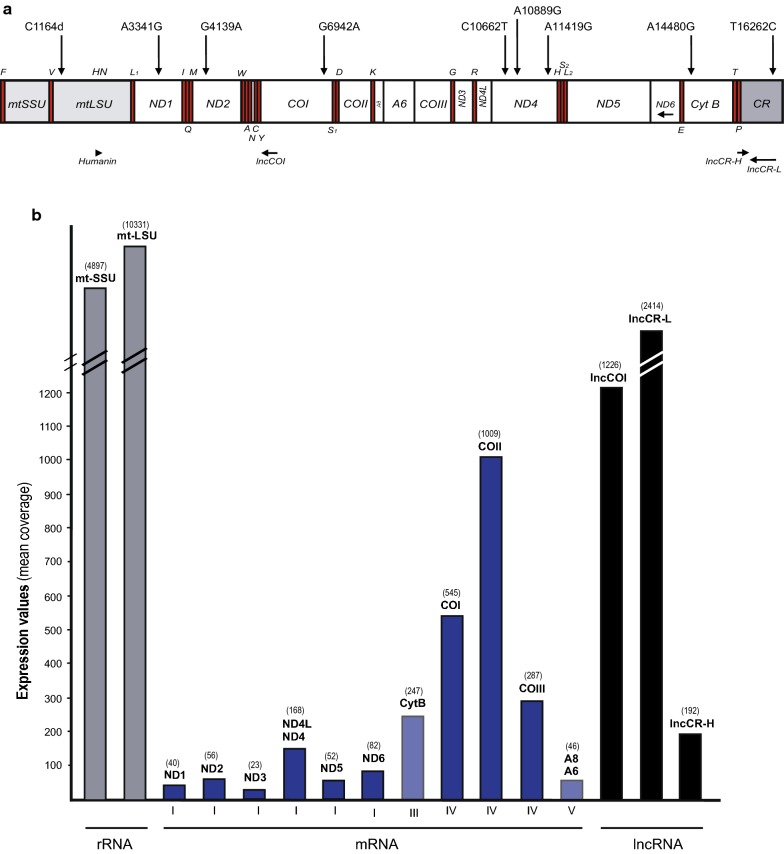
Mitochondrial genome organization and transcripts of *L. piscatorius*. **a** Mitochondrial genome presented as a linear map of the circular mtDNA. Single nucleotide polymorphisms in BF2 compared to BF1 are indicated above the gene map. Gene abbreviations: mtSSU and mtLSU, mitochondrial small- and large-subunit ribosomal RNA; ND1–6, NADH dehydrogenase subunit 1 to 6; COI-III, cytochrome oxidase subunit I to III; A6 and A8, ATPase subunit 6 and 8; Cyt B, cytochrome b; lncCR-H and lncCR-L, long non-coding RNAs coded by the control region (CR); lncCOI, long noncoding antisense RNA. tRNA genes are indicated by the standard one-letter symbols for amino acids. All genes are H-strand specific, except Q, A, N, C, Y, S_1_, E, P, ND6, lncCOI and lncCR-L (L-strand). **b** Histogram presentation of mean coverage expression values of mt-rRNAs, mRNAs, and lncRNAs based on Ion Torrent S5 total RNA sequencing

Complete mitochondrial genome sequences of two *L. piscatorius* specimens were determined using the Ion PGM and SOLiD5500 technologies (BF1; 2532 times mean coverage; MF994812; [13]) and the Illumina HiSeqX pair-end reads (BF2; 7643 times mean coverage; MN240767). The circular mtDNA possesses the conventional gene content and organization typical in vertebrates (Fig. 1a). Among the nine polymorphic sites between BF1 and BF2, seven were located in protein coding genes, representing both synonymous and non-synonymous amino acid substitutions (Additional file 1: Table S1).

Mitochondrial transcripts from *L. piscatorius* BF2 were generated by Ion S5 sequencing. About 145.2 million quality-filtered total RNA reads were obtained, including 510,484 reads (0.35%) unambiguously identified as mitochondrial transcripts when mapped to the BF2 mitochondrial genome. Several features were noted when inspecting the mitochondrial transcripts and correlating the expression values to specific mitochondrial gene regions (Fig. 1b): (1) reads from mt-rRNA gene transcripts were 10–100 times more abundant than protein coding transcripts. This observation is likely underestimated due to rRNA depletion of input RNA. (2) Of coding transcripts, cytochrome oxidase subunits were the most abundant, with NADH dehydrogenase subunits and ATPase subunits transcripts being much less abundant. (3) Highly abundant lncRNAs mapping to opposite strands within the mitochondrial CR and cytochrome oxidase I gene (COI) were noted. (4) Most mRNAs were polyadenylated and lacked 5′ and 3′ untranslated regions (UTRs) (Additional file 2: Table S2). A notable exception was the 75 nt 3′UTR of the COI mRNA (see below). Secondary structure predictions of *L. piscatorius* mt-SSU rRNA (Additional file 3: Figure S1) and mt-LSU rRNA (Additional file 4: Figure S2) showed typical fish mitochondrial features [7, 8]. Secondary structure predictions of all 22 tRNAs (Additional file 5: Figure S3) followed the general pattern of fish mitochondrial tRNAs [7].

#### *Non*-*canonical mitochondrial genes in* L. piscatorius

**Fig. 2:**
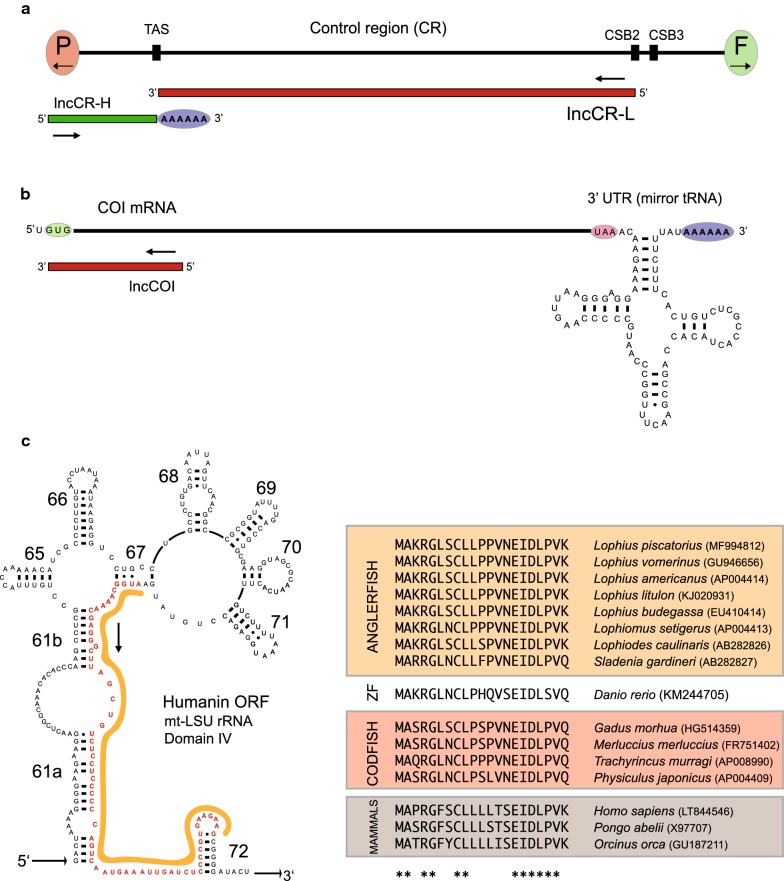
Non-canonical mitochondrial gene products in *L. piscatorius*. **a** Schematic view of CR and the long noncoding RNAs lncCR-L (approx. 620 nt) and lncCR-H (approx. 140 nt). P and F, tRNA^Pro^ and tRNA^Phe^ genes; TAS, termination associated sequence; CSB2 and 3, conserved sequence box 2 and 3. **b** Schematic view of the COI mRNA structure and lncCOI (178 nt). The translation initiation codon (GUG) and termination codon (UAA) are indicated. The 3′UTR contains a 75 nt mirror tRNA^Ser^ motif. **c** Left panel: Secondary structure diagram of the mt-LSU rRNA Domain IV of *L. piscatorius* with coding potential of a humanin-like peptide. See Additional file 4: Figure S2 for complete secondary structure diagram of mt-LSU rRNA. Right panel: Amino acid alignment of humanin-like peptides in anglerfish, zebrafish (ZF), codfish and mammals. Indicated ‘stars’ below the alignment represent conserved residues

The two CR specific lncRNAs (lncCR-H and lncCR-L), transcribed from opposite strands in a non-overlapping organization (Fig. 2a), have previously been reported in Atlantic cod [11, 12] and human [18]. The L-strand specific lncCR-L was found to be 30 times more abundant than the L-strand specific ND6 mRNA (Fig. 1b). The vertebrate mitochondrial COI mRNA is unusual due to the presence of a structured 3′UTR. We identified a polyadenylated COI mRNA containing a 75-nt 3′UTR in *L. piscatorius* (Fig. 2b). RNA-Seq data revealed a highly abundant 178 nt antisense RNA to the 5′ end of COI mRNA (Figs. 1b and 2b), which appeared novel among vertebrate mitochondrial lncRNAs and named lncCOI.

MDPs have been reported in vertebrates, and the best characterized is the humanin peptide [19]. The humanin gene is located within the mt-LSU rDNA locus. *L. piscatorius* contains a humanin-like open reading frame (ORF) in the mt-LSU rRNA Domain IV, at the exact same location as in Atlantic cod and human (Fig. 2c, left panel). Sequence analysis revealed the derived peptide sequence to be invariant within the *Lophius* genus, highly conserved among fishes, and well conserved between fish and mammals (Fig. 2c, right panel).

### Discussion

Here we provide the complete mitochondrial genome sequence and key features of the corresponding transcriptome of the anglerfish *L. piscatorius*. We found all canonical mitochondrial genes to be expressed. Mt-rRNAs were clearly more abundant than mRNAs. Two lncRNAs (lncCR-L and lncCR-H) mapped to the mitochondrial CR, a finding that corroborates recent reports of Atlantic cod and human cells [2, 18]. Interestingly, we identified a novel and highly abundant antisense RNA (lncCOI). Finally, we present feature support for the encoding of a humanin-like peptide within the mt-LSU rRNA.

Teleost fish mitochondria generate 10 mature mRNAs from a single primary transcript (HSP_2_) that subsequently are translated into 12 mitochondrial proteins in OxPhos complexes I, III, IV and V [2, 6]. Thus, the observed differences in transcript abundance may be explained by differential stability of individual mRNAs, and not by transcription initiation. Fish mitochondrial mRNAs contain no, or very short UTRs. A notable exception is the approximately 75-nt 3′UTR of the COI mRNA, which is conserved between fish species [2, 6] and mammals [20]. A study in rat showed that the nuclear miR-181c was regulating COI mRNA stability in heart tissue by 3′UTR binding [21]. A similar 75-nt 3′UTR was detected in the polyadenylated *L. piscatorius* COI mRNA. It is plausible, that the 3′UTR structure in *L. piscatorius* contributes to the COI mRNA stability.

A number of mitochondrial lncRNAs have been noted and characterized in vertebrates [reviewed in 2, 22, 23], but no lncRNA has so far been linked to COI gene sequences. Our observation of lncCOI appears novel among vertebrates. If the highly abundant lncCOI contributes to mRNA stability, translational regulation, or other mitochondrial roles is currently not known. We also detected two CR-specific lncRNAs (lncCR-L and lncCR-H) in *L. piscatorius*. lncCR-L corresponds to the 5′ end region of the LSP primary transcript and has been detected in Atlantic cod [6]. lncCR-L appears homologous to the 7S RNA reported in human mitochondria more than three decades ago [24], that was recently shown to be aberrantly expressed in human cancer cells [18]. Interestingly, lncCR-L was the most abundant non-ribosomal mitochondrial transcripts in *L. piscatorius*. lncCR-H, on the other hand, corresponds to the 3′ end region of the HSP_2_ primary transcript. It has been reported in Atlantic cod to be polyadenylated, to harbor a mirror tRNA, a noncoding intergenic spacer, and heteroplasmic tandem repeats [11, 12]. Similar to that of Atlantic cod, the *L. piscatorius* lncCR-H contains a mirror tRNA and a polyA tail. lncCR-L and lncCR-H may function as precursors for mitosRNAs [2], but their biological role has not been elucidated.

Reports in mammals conclude that the humanin peptide has important roles in cellular signaling [19, 25–27]. Previously we presented evidence supporting the encoding of humanin-like peptides in Domain IV of the mt-LSU rRNA in gadiform fishes [2], and similar features have recently been reported in avians [28]. Here we show that several anglerfishes, including all *Lophius* species where mtDNA sequences are available, possess humanin-like ORFs. How vertebrate humanin is translated is under debate, but different scenarios may be considered; (1) The humanin ORF is recognized in mt-rRNA by mitochondrial ribosomes and translated in mitochondria. This scenario is supported by a recent study in rat [26]. (2) Translation may also occur in cytosolic ribosomes, which would require mitochondrial export. Interestingly, a chimeric mt-LSU rRNA (lncRNA SncmtRNA) was reported to be expressed in human proliferating cells and localized in the cytoplasm and the nucleus [29, 30]. (3) Humanin may also be expressed from a nuclear copy of mt-LSU rRNA (Numt sequence). Studies from human cells provide support for the expression of nuclear-encoded humanin isoforms [31]. The latter scenario may explain why most, but not all, fish species have intact humanin-like ORFs in Domain IV.

### Conclusion

Our study provides a mitochondrial transcriptome resource from *L. piscatorius* heart muscle tissue. All mitochondrial genes were expressed, and different mRNAs had different abundances. Two lncRNAs mapped to the control region, we identified one novel lncRNA antisense to the COI mRNA, and the mt-LSU rRNA has the potential of coding a humanin-like peptide.

## Limitations

Mitochondrial RNA sequencing was performed in one tissue type in one individual and has to be considered as a *snapshot* of the mitochondrial transcriptome of *L. piscatorius*.

## Supplementary information

**Additional file 1: Table S1.** Polymorphic sites in the mitochondrial genome of *L. piscatorius* specimens BF1 and BF2.

**Additional file 2: Table S2.** 5′ and 3′ sequence features of *L. piscatorius* mitochondrial mRNAs derived from RNA-seq reads.

**Additional file 3: Figure S1.** Complete secondary structure diagram of *L. piscatorius* mitochondrial small subunit rRNA.

**Additional file 4: Figure S2.** Complete secondary structure diagram of *L. piscatorius* mitochondrial large subunit rRNA. Polymorphic site between BF1 and BF2 is indicated in Domain I. Low-level heteroplasmic sites in BF1 are indicated in Domains I and VI.

**Additional file 5: Figure S3.** Secondary structure diagram of *L. piscatorius* mitochondrial tRNAs. Anti-codon triplets and the non-template CCA are indicated.

## Data Availability

The RNA-seq raw sequencing data accession number at NCBI’s Sequence Read Archived (SRA) is SRS5181095. Accession numbers of mitogenomes are available from GenBank under the Accession Number MF994812 (BF1) and MN240767 (BF2).

## Abbreviations

CR: control region
lncRNA: long noncoding RNA
LSU: large subunit
MDP: mitochondrial-derived peptide
mitosRNA: mitochondrial small RNA
mtDNA: mitochondrial DNA
OxPhos: oxidative phosphorylation
SSU: small subunit
UTR: untranslated region
