# Supplementary material for: The mitochondrial transcriptome of the anglerfish Lophius piscatorius
Source: BMC Res Notes. 2019 Dec 10;12:800. doi: 10.1186/s13104-019-4835-6 (PMC6905026; doi:10.1186/s13104-019-4835-6)
Supplement: Supplementary file 1 — Additional file 1: Table S1. Polymorphic sites in the mitochondrial genome of L. piscatorius specimens BF1 and BF2. [file 13104_2019_4835_MOESM1_ESM.pdf]

Additional file 1: Table S1. Polymorphic sites in the mitochondrial genome of *L. piscatorius* specimens BF1 and BF2

Polymorphic site [1]	Gene region [2]	Codon	Amino acid
C1164d	mtLSU	-	-
A1165C (2.6%) [3]	mtLSU	-	-
T1168A (1.9%) [3]	mtLSU	-	-
A2678G (1.3%) [3]	mtLSU	-	-
G2679A (1.3%) [3]	mtLSU	-	-
A3341G	ND1	AGC to GGC	S to G
G4139A	ND2	AGC to AAC	S to N
G6942A	COI	GAG to GAA	E (synonymous)
G9120C (1.4%) [3]	COIII	TGG to TCG	W to S
C10662T	ND4	ACT to ATT	T to I
A10889G	ND4	AAC to GAC	N to D
A1141G	ND4	TGA to TGG	W (synonymous)
A14480G	CytB	GCA to GCG	A (synonymous)
T16158C (1.0%) [3]	CR	-	-
C16160T (1.3%) [3]	CR	-	-
T16262C	CR	-	-

Notes: [1] Positions are according to the reference sequence in specimen BF1 (MF994812). [2] mtLSU, mitochondrial large subunit ribosomal RNA; ND1, NADH dehydrogenase subunit 1; COI, cytochrome c oxidase subunit I; COIII, cytochrome c oxidase subunit III; ND4, NADH dehydrogenase subunit 4; CytB, cytochrome B. [3] Low-level substitution heteroplasmy detected in the BF1 specimen by SOLiD ligation sequencing at 2227 times mtDNA coverage [Dubin et al. 2017]. Reference: Dubin A, Jørgensen TE, Jakt LM, Moum T, Johansen SD: The mitochondrial genome of the European Anglerfish Lophius piscatorius express low-level substitution heteroplasmy. Ann Mar Biol Res. 2017;4:1019.
