# Supplementary material for: The mitochondrial transcriptome of the anglerfish Lophius piscatorius
Source: BMC Res Notes. 2019 Dec 10;12:800. doi: 10.1186/s13104-019-4835-6 (PMC6905026; doi:10.1186/s13104-019-4835-6)
Supplement: Supplementary file 2 — Additional file 2: Table S2. 5′ and 3′ sequence features of L. piscatorius mitochondrial mRNAs derived from RNA-seq reads. [file 13104_2019_4835_MOESM2_ESM.pdf]

Additional file 2: Table S2. 5' and 3' sequence features of *L. piscatorius* mitochondrial mRNAs derived from RNA-seq reads

mRNA [1]	5' end [2]	3' end [3]
ND1	**AUG** AUC UCA	CAA AUA **UAA** aaaaaaaaaa
ND2	**AUG** AAC CCC	GCC CUU **Uaa** aaaaaaaa
ND3	**AUG** AAC UUA	GCC GAA **Uaa** aaaaaaaa
ND4L/ND4	**AUG** ACC CCC	AGC AUA **Uaa** aaaaaaaa
ND5	**AUG** CAC CCU	n.d.
ND6	**AUG** ACU UAU	n.d.
CytB	n.d.	AAA UUA **UAG** aaaaaaaaaa
COI	U **GUG** GCA AUC	ACC CGU **UAA** ACAAGAAAGGAGGGAAUUGAACCCCCGUAACCCGGUUUCAAGC CGACCACAUCACCGCUCUGUCACUUUCUUUAUaaaaaaaaaa
COII	n.d.	GAC GCU **Uaa** aaaaaaaa
COIII	n.d.	GGC UCA **UAa** aaaaaaaaaa
A8/A6	U **AUG** CCC CAA	AAC GUA **UAA** aaaaaaaaaa

Notes: [1] Eleven mitochondrial mRNAs: ND1-6, NADH dehydrogenase subunits 1-6 mRNAs; CytB, cytochrome B subunit mRNA; COI-III, cytochrome c oxidase subunits I-III; A8/A6, ATPase 8/6 mRNA. [2] The 5' sequence contains the three first codons. Start codon (AUG/GUG) is under-lined and in bold. Defined 5' end not detected (n.d.) due to low coverage. [3] The 3' sequence contains the three last codons. Termination codon (UAG/UAA) is under-lined and in bold. Non-template adenosines are indicated by lower case 'a'. Defined polyA-tail not detected (n.d.).
