# Supplementary figures and images for: The mitochondrial transcriptome of the anglerfish Lophius piscatorius
Source: BMC Res Notes. 2019 Dec 10;12:800. doi: 10.1186/s13104-019-4835-6 (PMC6905026; doi:10.1186/s13104-019-4835-6)

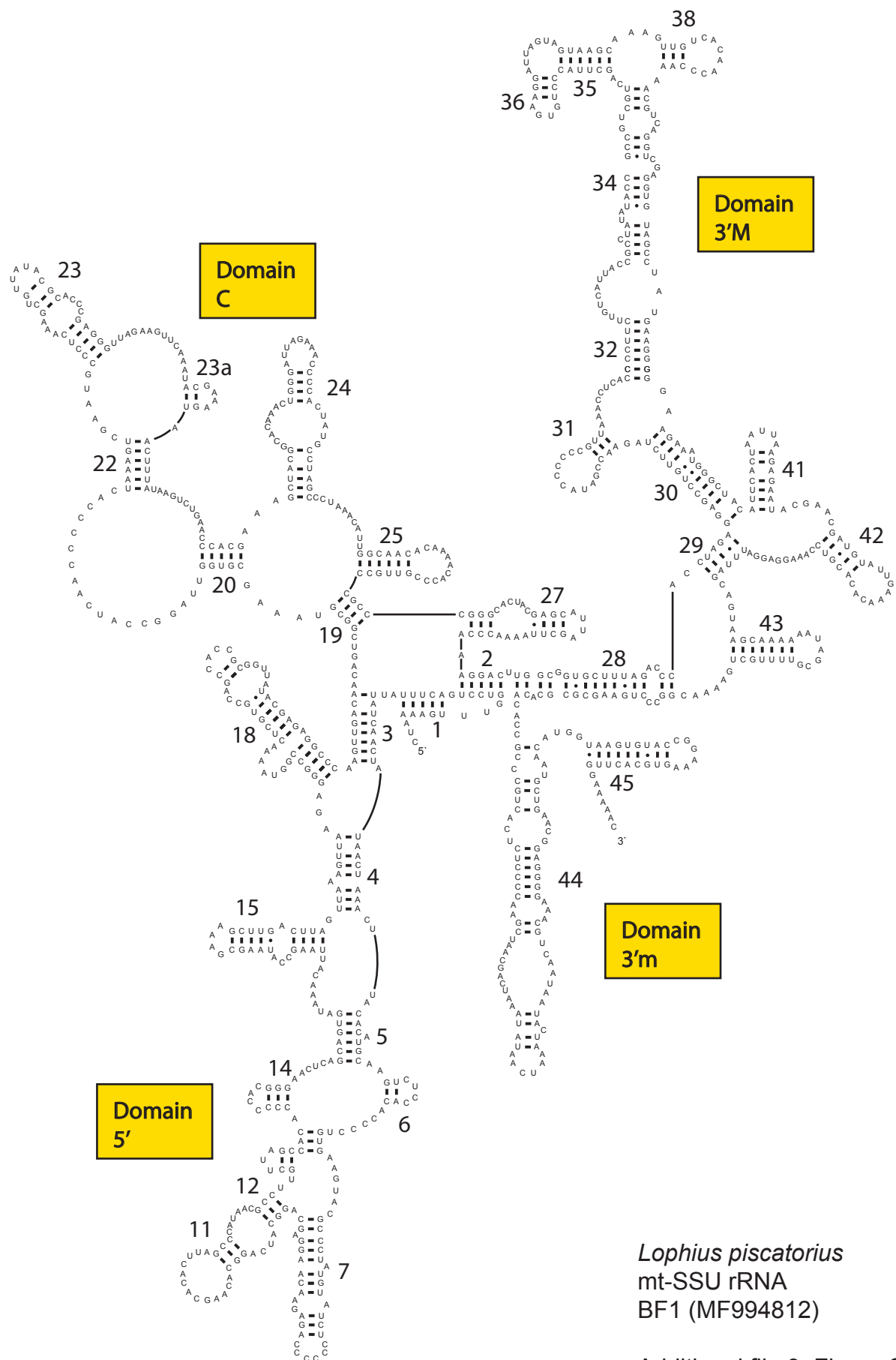

Additional file 3: Figure S1

Supplement: Supplementary file 3 — Additional file 3: Figure S1. Complete secondary structure diagram of L. piscatorius mitochondrial small subunit rRNA. [file 13104_2019_4835_MOESM3_ESM.pdf]

Additional file 5: Figure S3a

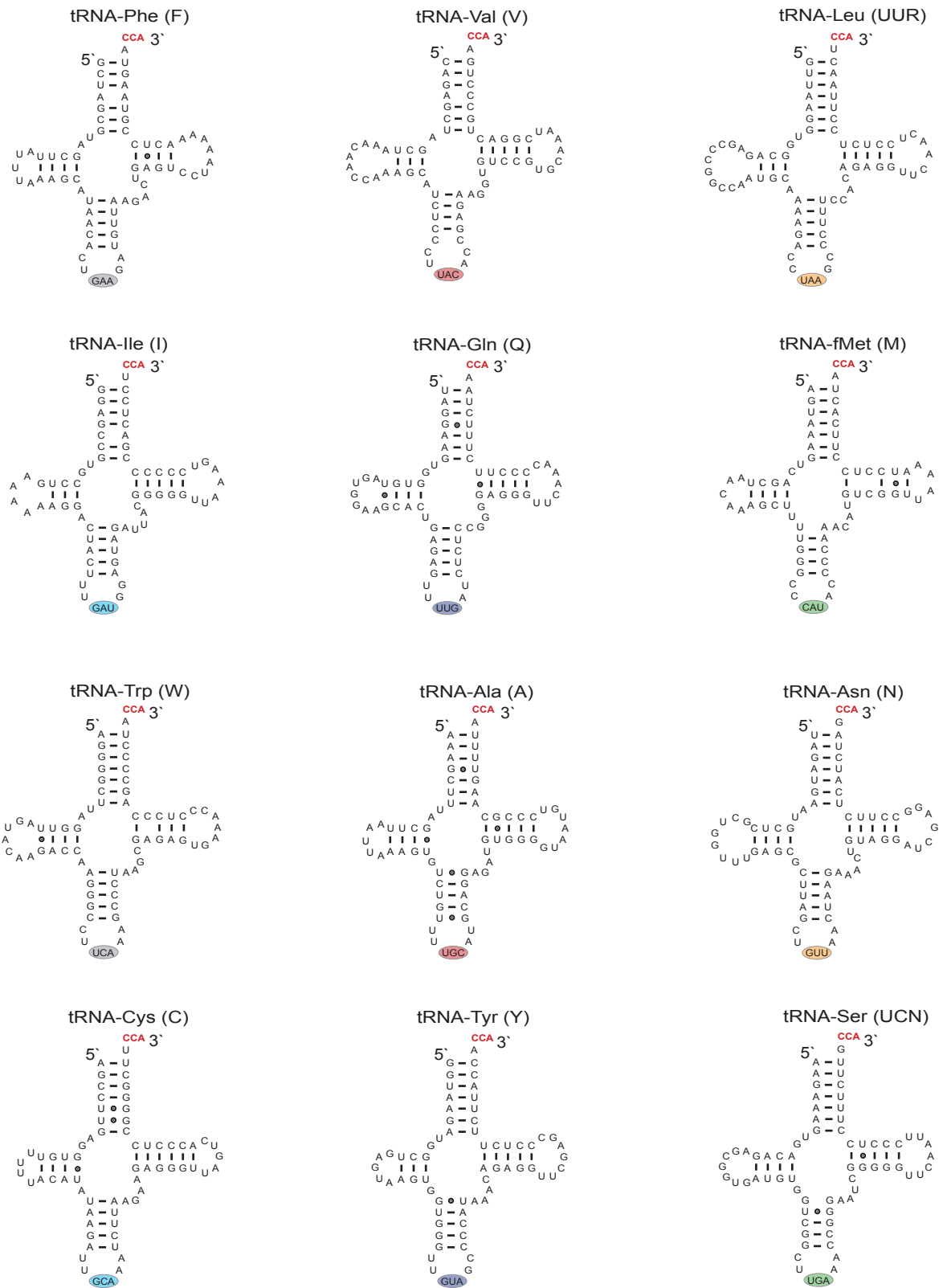

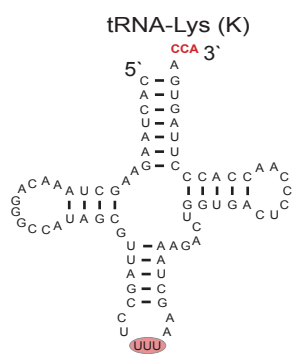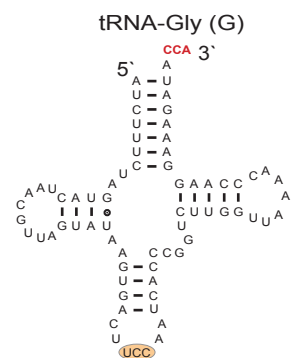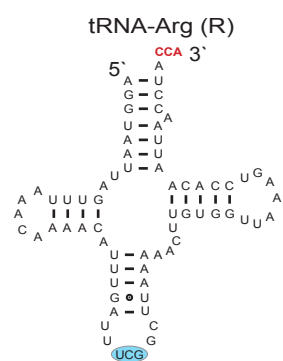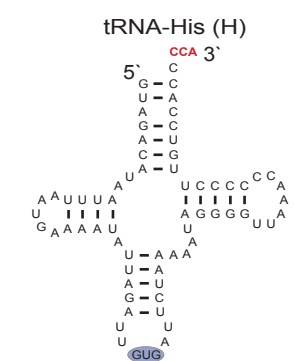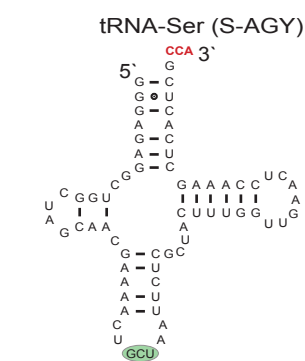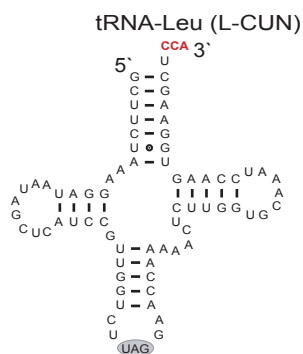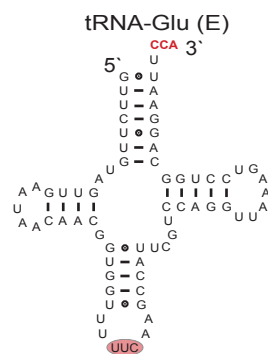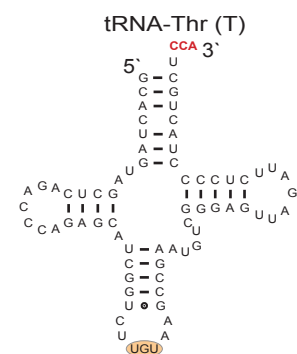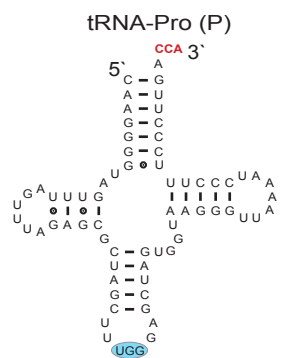

Supplement: Supplementary file 5 — Additional file 5: Figure S3. Secondary structure diagram of L. piscatorius mitochondrial tRNAs. Anti-codon triplets and the non-template CCA are indicated. [file 13104_2019_4835_MOESM5_ESM.pdf]
