# Supplementary material for: The mitochondrial transcriptome of the anglerfish Lophius piscatorius
Source: BMC Res Notes. 2019 Dec 10;12:800. doi: 10.1186/s13104-019-4835-6 (PMC6905026; doi:10.1186/s13104-019-4835-6)
Supplement: Supplementary file 4 — Additional file 4: Figure S2. Complete secondary structure diagram of L. piscatorius mitochondrial large subunit rRNA. Polymorphic site between BF1 and BF2 is indicated in Domain I. Low-level heteroplasmic sites in BF1 are indicated in Domains I and VI. [file 13104_2019_4835_MOESM4_ESM.pdf]

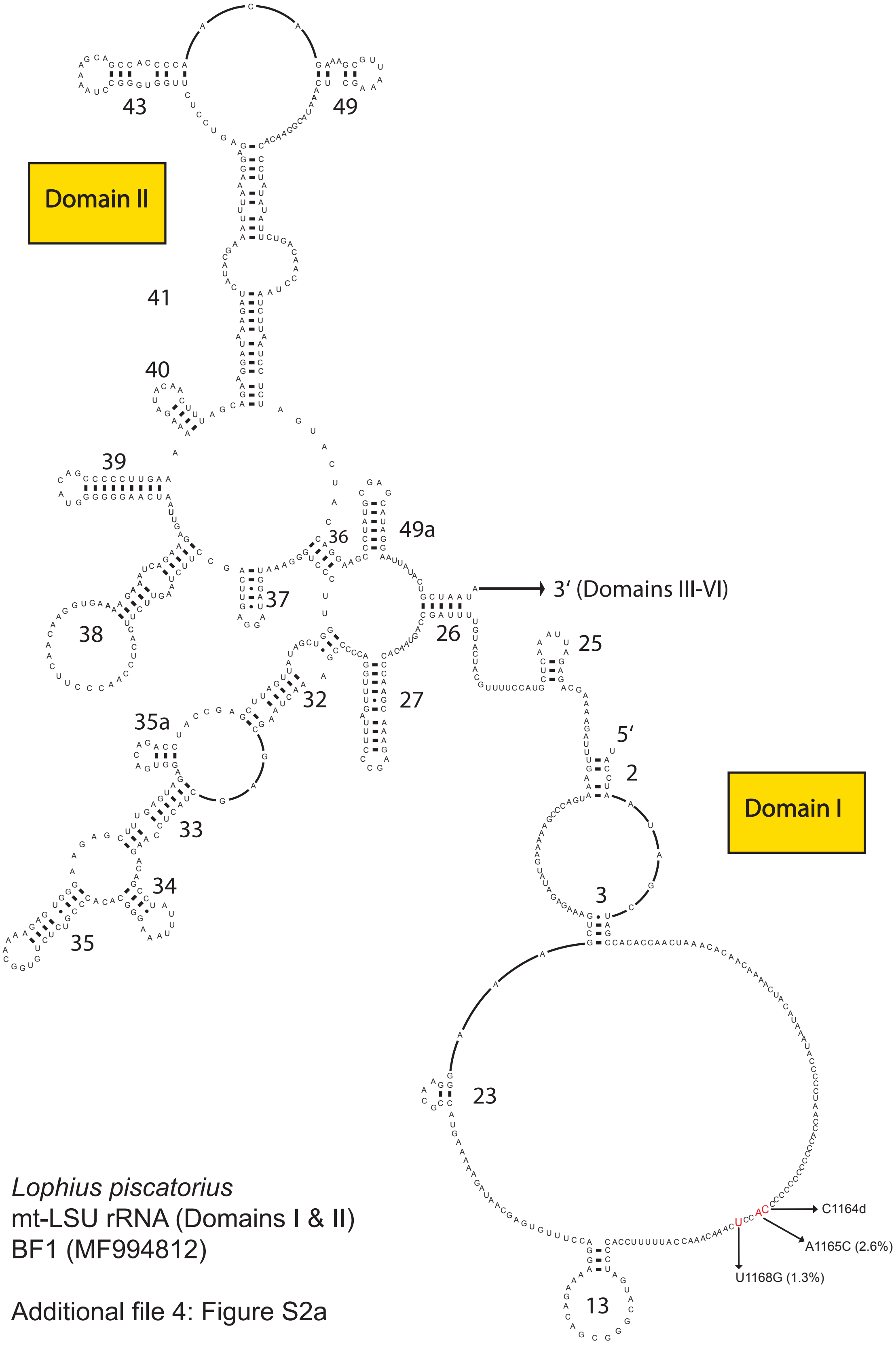

Lophius piscatorius
mt-LSU rRNA (Domains I & II)
BF1 (MF994812)

Additional file 4: Figure S2a

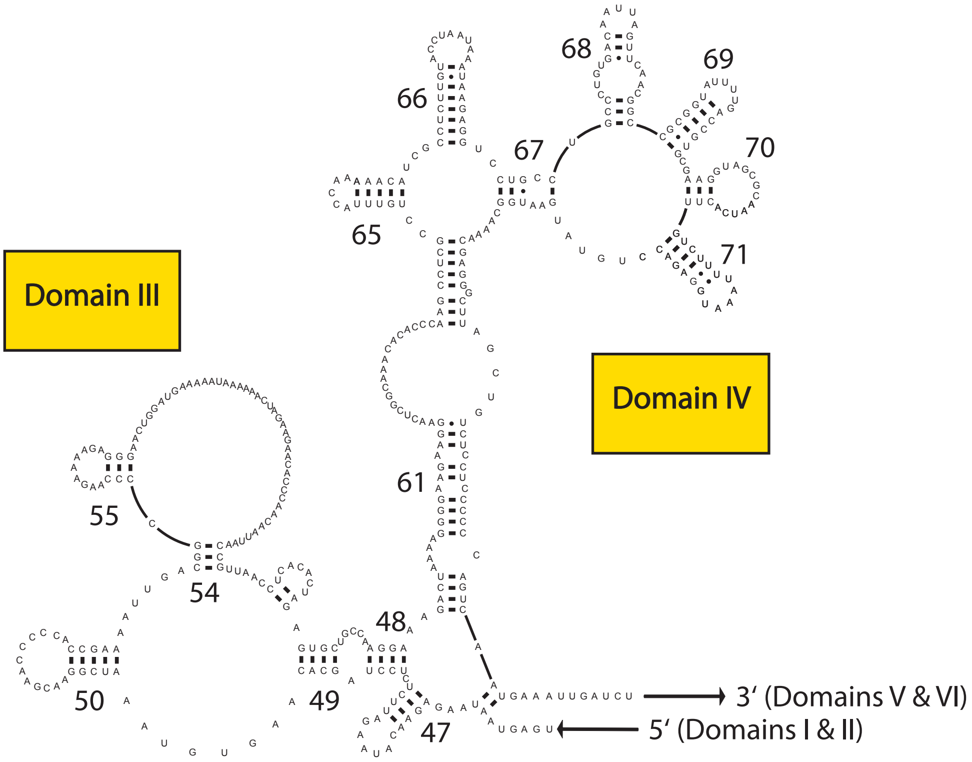

Lophius piscatorius
mt-LSU rRNA (Domains III & IV)
BF1 (MF994812)

Additional file 4: Figure S2b

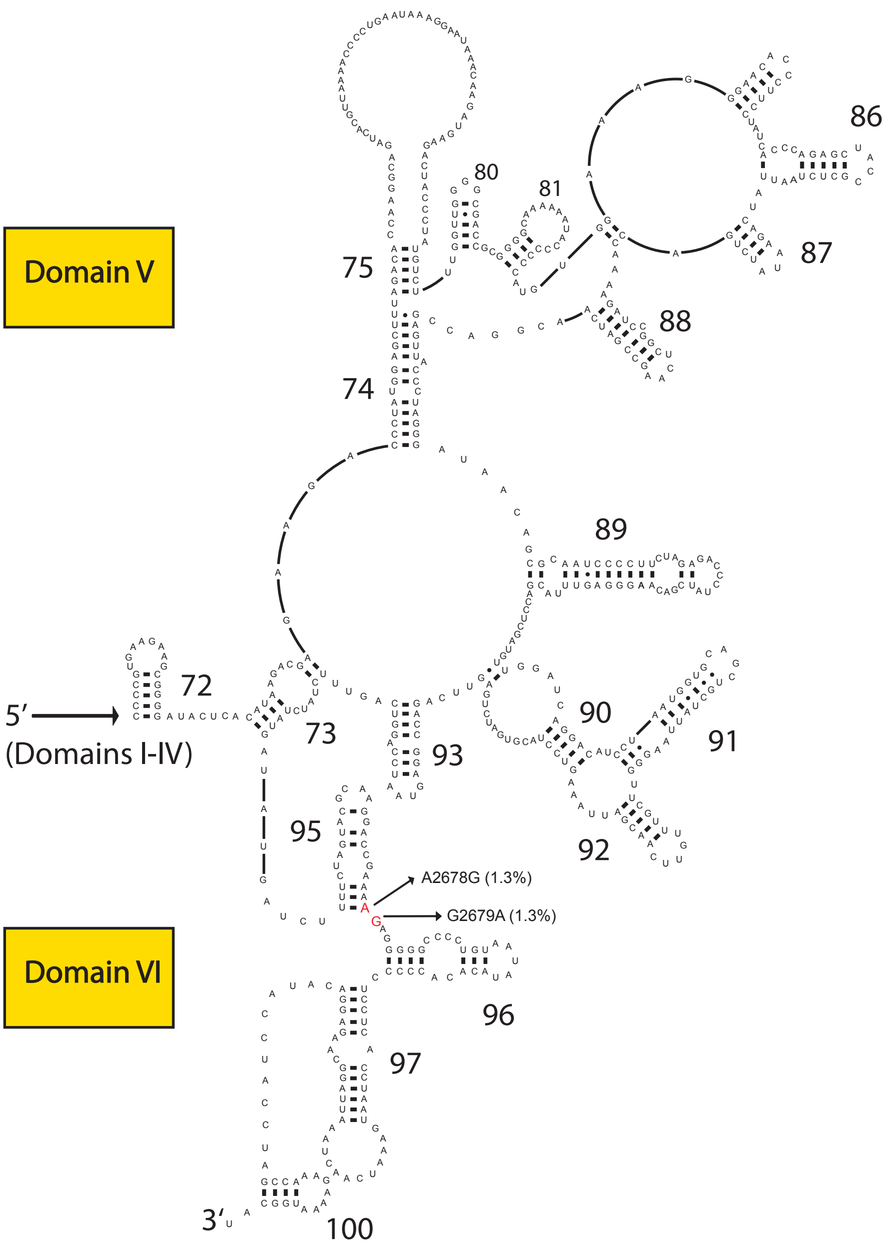

Lophius piscatorius
mt-LSU rRNA (Domains V & VI)
BF1 (MF994812)

Additional file 4: Figure S2c
